# Atomic‐Level Strain Sensing and Piezoresistance Effect in a 1D Single‐Atom Chain

**DOI:** 10.1002/advs.202500553

**Published:** 2025-04-26

**Authors:** Zhi Qu, Wenqi Zhang, Shuideng Wang, Donglei Chen, Yiqing Yao, Mingxing Cheng, Lixin Dong

**Affiliations:** ^1^ Department of Biomedical Engineering City University of Hong Kong Hong Kong 999077 P. R. China

**Keywords:** dynamic nanostructure, nano piezoresistance effect, nano strain sensing, single‐atom chain

## Abstract

Small variations in interatomic distances have a substantial impact on the physical and chemical properties of nanomaterials. Investigating these effects offers a deeper understanding of the mechanisms governing the behavior of nanomaterials and nanostructures, providing foundations for the design and optimization of novel functional materials. However, the impact of strain in single‐atom structures on piezoresistance and electronic transport properties remains unclear. This study focuses on a 1D, dynamic functional nanostructure that uses interatomic distance variations for lattice‐level strain sensing. This silver (Ag) atom chain shows a high stability at room‐temperature and an exceptional piezoresistance coefficient, enabling the detection of structural changes at atomic radius scale with high sampling frequencies. It is considered that this strong piezoresistivity is due to the impact of interatomic distance on electron scattering and transport mechanisms. The density functional theory simulations of electron transport reveal that variations in interatomic distance significantly influence the relaxation time of electron scattering and the effective electron mass, thereby modulating the characteristics of electron transport. This 1D dynamic nanostructure has the potential to address the low time resolution limitations of transmission electron microscopy (TEM), enhancing its capabilities for in situ characterization and multi‐physical‐field sensing. This study provides experimental evidence for insights into atomic scale piezoresistivity and underlying mechanisms.

## Graphical abstract

The atomic‐scale piezoresistance effect of the Ag single‐atom chain is explored using strain characterization and electrical measurement. Single‐atom chain has the potential to serve as a strain sensor with atomic‐level precision, overcoming the challenge of low temporal resolution in a transmission electron microscope. The theoretical explanation is also provided for the changes in interatomic distance and electronic transport properties of this structure.

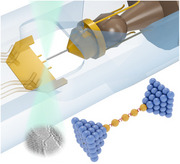

## 1 Introduction

Nanofunctional structures can respond to external stimuli or spontaneous conditions, facilitating the transmission and enhancement of interactions between external physical fields and matter in a wide range of classical and quantum applications, such as sensing, catalysis, imaging, and communication.^[^ ^1^, ^2^, ^3^, ^4^ ^]^ Despite their often simple and ingenious designs, these structures can achieve highly complex and efficient functionalities through structural or dynamic transformations.^[^ ^5^ ^]^ Beyond the intrinsic physical and chemical properties of the constituent materials, the interatomic spacing plays a pivotal role in determining their functionalities.

Interatomic spacing significantly influences the mechanical properties of nanostructures, including elastic modulus, strength, and hardness.^[^ ^6^ ^]^ In nanomaterials, the high proportion of grain boundaries relative to the overall structure results in reduced interatomic distances, which hinder dislocation mobility and suppress grain boundary sliding and dislocation propagation. This phenomenon, exemplified by grain boundary strengthening and dislocation pinning, substantially enhances material strength and hardness.^[^ ^7^, ^8^, ^9^ ^]^ Understanding how variations in interatomic spacing affect mechanical performance is essential for the design, fabrication, and application of nanodevices and reinforced composites.^[^ ^10^, ^11^ ^]^ Additionally, variations in interatomic spacing significantly influence the local environment of active atoms, which in turn affects catalytic chemical reactions.^[^ ^12^, ^13^, ^14^, ^15^ ^]^ The bond length between active centers and coordinating atoms is a key parameter determining catalytic activity.^[^ ^16^, ^17^, ^18^ ^]^ Strain‐induced compression or elongation of these bonds can modify molecular adsorption modes, significantly affecting catalytic performance.^[^ ^19^, ^20^ ^]^

Transformations in interatomic spacing within nanostructures significantly affect their optical properties, including absorption, emission, and reflection. Atomic optical antennas, which are essentially resonantly excited atomic optical dipoles, generate both propagating and evanescent electromagnetic fields. As the near‐field components become more pronounced with decreasing interatomic spacing, these structures demonstrate a substantial potential for near‐field intensity enhancement. Consequently, precise control of interatomic spacing within atomic optical antennas facilitates ultra‐efficient field enhancement.^[^ ^21^, ^22^, ^23^ ^]^

Changes in interatomic spacing also alter the electronic structure of materials, including band structure and electronic density of states, potentially affecting properties such as conductivity and bandgap.^[^ ^24^, ^25^, ^26^, ^27^, ^28^, ^29^ ^]^ External stress can distort lattice structures, introducing or amplifying scattering centers such as lattice defects, impurities, and interfaces. This leads to more frequent electron‐scattering events during transport, thereby influencing the electrical conductivity of crystalline structures.^[^ ^30^, ^31^ ^]^ Understanding the relationship between interatomic spacing variations and electrical properties is vital for guiding the design of nanostructures. However, direct characterization of the connection between in situ atomic‐level strain and electrical properties remains limited, and multiple mechanisms have been proposed.

This study conducts in situ strain and electrical characterization of 1D single‐atom chain, demonstrating the phenomena and mechanisms of macroscopic piezoresistive effects at the single‐atom level. It also highlights the significant differences between atomic‐scale and macroscopic piezoresistive effects. Beyond investigating the giant piezoresistive effect exhibited by single‐atom chain, this work also discusses and validates the potential of nanowires (not limited to atomic‐scale structures) as in situ dynamic sensing functional structures based on the piezoresistive effect. As described earlier, most studies focus on the conductance of quantum point contacts, short chains formed by a few atoms, or nanowires at the micron or submicron scale, without conducting direct in situ experiments and analyses on the piezoresistive effect at the atomic scale. In this study, using a dual spherical aberration corrected (Cs)‐TEM environment, we employed a nanomanipulator to in situ exfoliate long single‐atom chains on pristine crystalline surfaces, ensuring excellent cleanliness. The atomic‐scale piezoresistive phenomenon was observed in real time, and the piezoresistive effect of single‐atom chains was analyzed. Combining density functional theory (DFT) simulations of electron transport, we elucidated the mechanism of the piezoresistive behavior of atom chains, providing both experimental and theoretical insights into the piezoresistive effect at the atomic scale.

At the atomic level, this study demonstrates that atom chain/nanowire structures have the potential to serve as in situ dynamic sensing functional structures. In the future, atomic chains/nanowires could enable high‐precision, wide‐range, and high‐sampling‐rate strain sensing at the micro‐ and nanoscale. The strain‐sensitive atomic chain/nanowire structures can effectively compensate for the low sampling rate issue associated with vision‐based sensing in micro‐ and nanoscale characterization. Our study conducted at the minimal scale within the classical theoretical framework, provides both experimental and theoretical foundations for the future application of nanowire structures in micro‐ and nanoscale strain sensors. It broadens the theoretical support for the piezoresistive effect in nanoscale strain sensors, further confirming its significant application potential and offering new insights into in situ strain and electrical characterization of nanomaterials and structures.

## 2 In Situ Strain Characterization of Single Sliver Atom Chains

In an in situ characterization system equipped with a double Cs‐TEM, the sample region at the front end of a single‐tilt sample holder accommodates a fixed sample on one side and a triaxial nanomanipulator driven by lead zirconate titanate piezoelectric ceramics on the other. The manipulator is connected via a spherical joint to a tungsten (W) tip with a small amount of Ag nanofilm structure at its apex. Serving as the end‐effector of the in situ nanomanipulation system, the W tip manipulates with three degrees of freedom and achieves sub‐angstrom positioning precision, enabling in situ strain manipulation of nanocrystals, including tension, compression, shearing, and twisting.

At room‐temperature, the nanomanipulator positions the W tip near the edge of a thin Ag nanocrystal located on a semi‐copper mesh grid. The manipulator incrementally controls the approach of the Ag nanocrystal (with a thickness below 50 nm) on the W tip to make effective contact with the Ag nanocrystal on the copper grid edge, ensuring alignment in the thickness direction. During the entire contact process, a low, constant bias voltage is applied across the two ends of the sample. Due to the low diffusion barrier of single metal atoms on metallic surfaces (generally less than 1 eV), thermal activation at room‐temperature allows Ag atoms to overcome this barrier and diffuse along the metal crystal surface.^[^ ^32^, ^33^ ^]^ Through atomic diffusion, Ag atoms on the surfaces of the contact region combine to form a unified new structure. The form in which atoms exist on the crystal surface directly impacts diffusion efficiency, making it essential for a clean crystal surface to have only a limited number of independent Ag atoms. The formation of such relatively independent Ag atoms requires additional energy, which in this study is provided by locally applied external electric fields and mechanical manipulation. Subsequently, the manipulator brings the Ag crystals on both sides into proximity. Under the combined effects of the local electric field and van der Waals forces, several Ag atoms on both surfaces bind to form a relatively stable structure.

**Figure 1:**
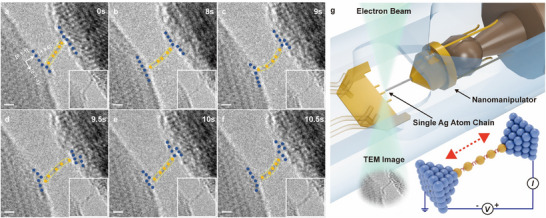
a–f) Time‐sequence series of TEM of deformation process of silver atom chain. The crystal orientation is [0 1–1] and the space of (−1 0 0), is 0.29 nm. A bias voltage of 0.5 V was applied between Ag electrodes. g) In situ single‐atom chain manipulation diagram.

The manipulator then separates the two Ag crystal surfaces at a sub‐nanometer step size. In the localized point‐contact region, stable metallic bonds formed between Ag atoms result in a stable 1D single‐atom chain during the separation process. **Figure** **1** presents a time series of high‐resolution TEM (HRTEM) images capturing the tensioning process of the Ag single‐atom chain. The left‐side crystal maintains effective contact with the semi‐copper mesh, while the right‐side crystal is attached to the W tip, with both Ag crystals aligned along the [0 1–1] orientation and an interplanar spacing of 0.29 nm for the (−1 0 0) crystal planes. After tensioning the atom chain to a length of five atoms, the interplanar spacing of both ends remains relatively stable. The two Ag crystals at the ends of the chain can be approximated as infinitely large Ag plates, across which a 0.5 V bias voltage is applied.

**Figure 2:**
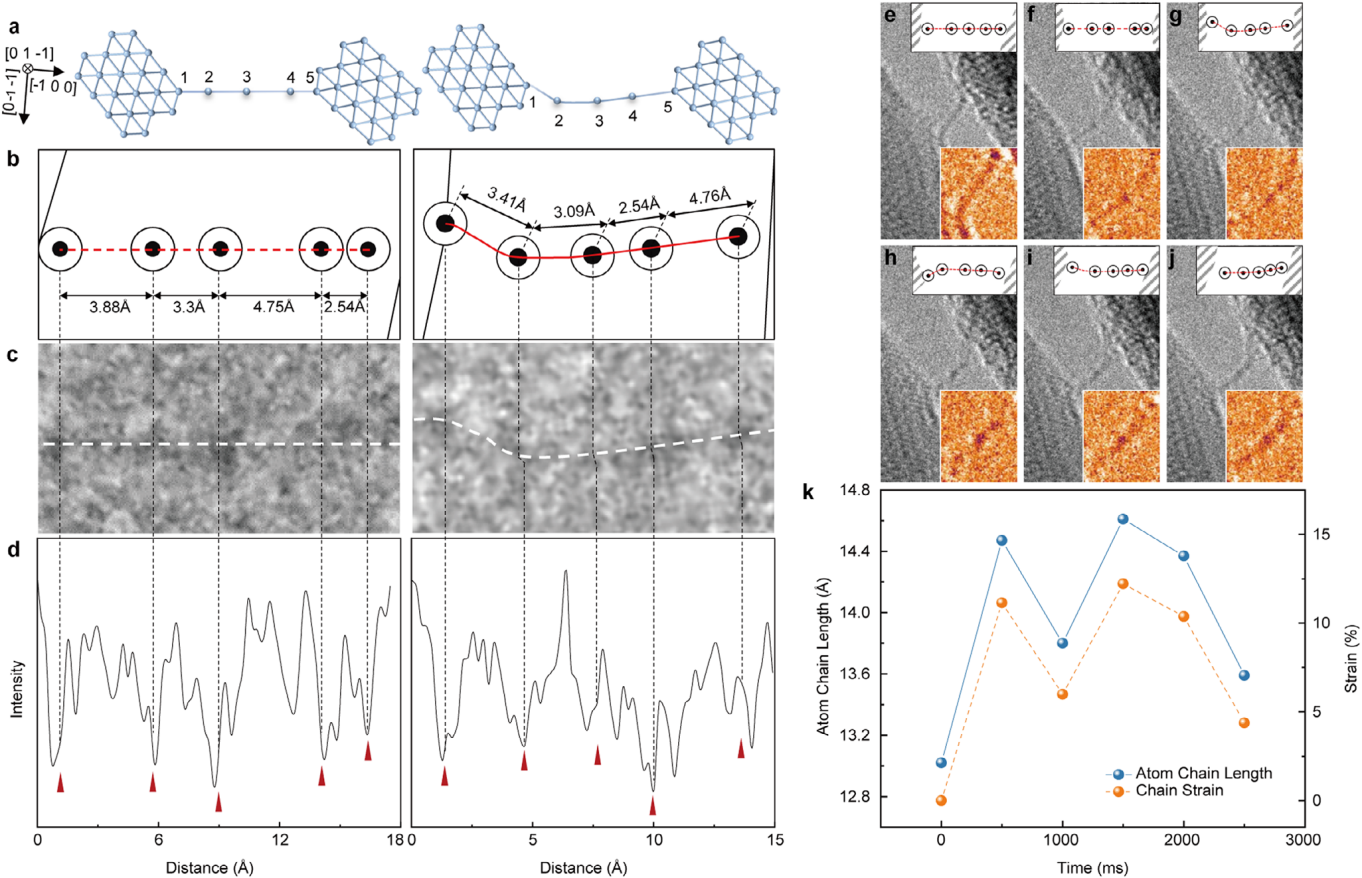
The distribution of atomic positions within the chain is determined based on the grayscale gradient and distribution obtained from HRTEM in situ characterization, HRTEM with pseudo‐color map, and the variation of atom chain length over time. a) Schematic diagram of the contact and structure of the atom chain at both ends. b) Atomic positions and spacing diagram within the atom chain determined based on the grayscale intensity gradient and distribution. c) In situ high‐resolution local image of atom chain. d) Average grayscale distribution curves along the axis of the atom chain. e–j) HRTEM with a pseudo‐color map of keyframes (with atom position). k) Changes in the length and strain of the Ag single‐atom chain over time.

In this process, the first Ag atom at the fixed end remains stationary, while the last Ag atom at the free‐moving end begins to pull the entire atom chain as the manipulator advances with sub‐angstrom precision. During this manipulation, the crystal orientations at the contact regions of both ends remain unchanged, but the positions of Ag atoms within the chain shift, leading to a significant deformation. To analyze the strain state of the single‐atom chain, it is essential to determine the number and positions of atoms in the chain. For each keyframe, multiple parallel translation lines along the atom chain's central axis (indicated by the white dashed line in **Figure** **2**c) were extracted to measure the grayscale intensity distribution. The *x*‐coordinate corresponding to the maximum peak in the single‐peak regions of the average intensity distribution curve was taken as the atomic center. For double‐peak regions, the *x*‐coordinate of the point with the maximum gradient between the two peaks was selected. The grayscale intensity distribution curves are illustrated in Figure 2d, and the determined atomic positions are shown in Figure 2b. Using the above method, the grayscale intensity distributions of the atom chain in all keyframes in Figure 1 were analyzed to identify atomic positions. Additionally, crystallographic characterization was performed to confirm that the Ag crystal orientation in the contact regions at both ends of the atom chain remained [0 1–1] throughout the entire strain process. A schematic model of the contact regions and atom chain structure is shown in Figure 2a.

From its formation to repeated tensioning, deformation, and eventual rupture, the atom chain experienced atomic‐level positional shifts under the combined effects of external stress and internal metallic bonding forces. The entire process lasted 30 s, during which the atom chain exhibited excellent structural stability. For the six keyframes in Figure 1, the position distributions of atomic centers over time were statistically analyzed. By measuring the distance between the atomic centers at both ends of the chain, the total chain length was determined, and interatomic spacing was analyzed frame‐by‐frame to calculate atomic‐level strain in the single‐atom chain, as shown in Figure 2k. Notably, the atoms at both ends of the chain were not entirely isolated on the crystal surfaces. A certain number of atoms remained on the crystal surfaces during the contact‐separation process, forming metallic bonds with the chain terminal atoms. Additionally, van der Waals forces were significantly enhanced by the increased number of nearby surface atoms. As illustrated in Figure 1, the surface of the right‐side Ag crystal contains a greater number of atoms interacting with the terminal atom of the chain, leading to stronger bonding at the right end. The atoms on the right side of the chain are more densely packed and have smaller interatomic spacings, resulting in a nonuniform distribution of atomic arrangement within the chain. Variations in interatomic spacing and their uneven distribution manifested as strain within the atom chain, which also affected electron transport along the chain. This directly altered electrical current, a phenomenon attributed to the atomic scale piezoresistance effect.

## 3 In Situ Electrical Characterization and Piezoresistance Effect Analysis During Strain Process in Single‐Atom Chain

Under external stress, the lattice‐level strain induced in the Ag single‐atom chain directly influences the carrier transport behavior, which is macroscopically manifested as a change in current.^[^ ^34^, ^35^ ^]^ This phenomenon represents a direct manifestation of the piezoresistance effect at the atomic scale. Through in situ electrical characterization of the strain process, the correlation between lattice strain and chain conductance is systematically analyzed, providing a quantitative description of the piezoresistance effect at the lattice scale. Additionally, the piezoresistance coefficient of the atom chain sensor is calibrated to further investigate the mechanism and feasibility of lattice‐scale sensing. Before analyzing the high‐resolution characterization, electrical characterization, and piezoresistive effect of the atom chain, it is necessary to first clarify the impact of the equivalent capacitance formed by the crystals on both sides of the atom chain on electron transport within the chain, as well as whether it affects the in situ sampling data of the chain current. A detailed explanation of three factors that influence the electrical properties of the atom chain in this study is provided below (The detailed calculation process can be found in the Supporting Information).

1) From the perspective of the equivalent capacitance size of the crystals on both sides of the silver atom chain. In the absence of the atom chain, the entire system can be considered a nanoscale capacitor. Here, we calculate the possible maximum capacitance value. Assuming the entire probe tip actuator serves as the capacitor, the maximum capacitor area (*A*) is given by *A*  =  40nm × 100nm  =  0.004µm^2^. The spacing is uniformly taken as the minimum distance of 1.2 nm (although only a very small region actually has this spacing). According to the classical capacitance formula, the maximum capacitance (*C_A_*) between the two crystal plates is ≈ *C_A_* =  2.95 µF cm^−2^. Although the distance between the two crystal planes is small, the overall area of the crystal planes (equivalent capacitor plates) is also very limited. Consequently, the equivalent capacitance of the entire system is not significantly large, and its impact on the electron transport characteristics of the atom chain system is relatively minor.

2) From the perspective of charge accumulation and local electric field effects potentially inducing electron tunneling in the adjacent crystals (capacitor). A significant tunneling current only appears when the tunnel junction spacing is below 0.7 nm. In this experiment, the applied voltages are 0.5 and 0.25 V, the average length of the atom chain is 1.36 nm, and the electrode spacing is above 1.2 nm. This indicates that in the single‐atom chain system of this study, tunneling phenomena caused by local charge accumulation and electric field effects have a negligible impact on the measurement of chain current.

3) From the perspective of the Coulomb blockade effect. Due to the discreteness of charge and the capacitive effect, electron transport is hindered when electrons need to overcome the energy required for charge redistribution, leading to blockade or significant suppression of current flow. Here, we calculate the coulomb energy as *E_C_* =  2.713 meV. When the applied voltage *V* > *V_thresh_*, a significant Coulomb blockade effect does not occur. In this study, the applied bias voltages are 0.5 and 0.25 V, satisfying *V_bias_* > 2.713 mV, which is far greater than the threshold voltage. Additionally, the in situ characterization was performed at room‐temperature. Therefore, the Coulomb blockade effect induced by capacitance can be considered negligible. The above explanations demonstrate that the equivalent nanoscale capacitance formed by this structure has a very limited impact on the current in the single‐atom chain under the applied bias voltage, electrode spacing, and temperature conditions set in this study.

**Figure 3:**
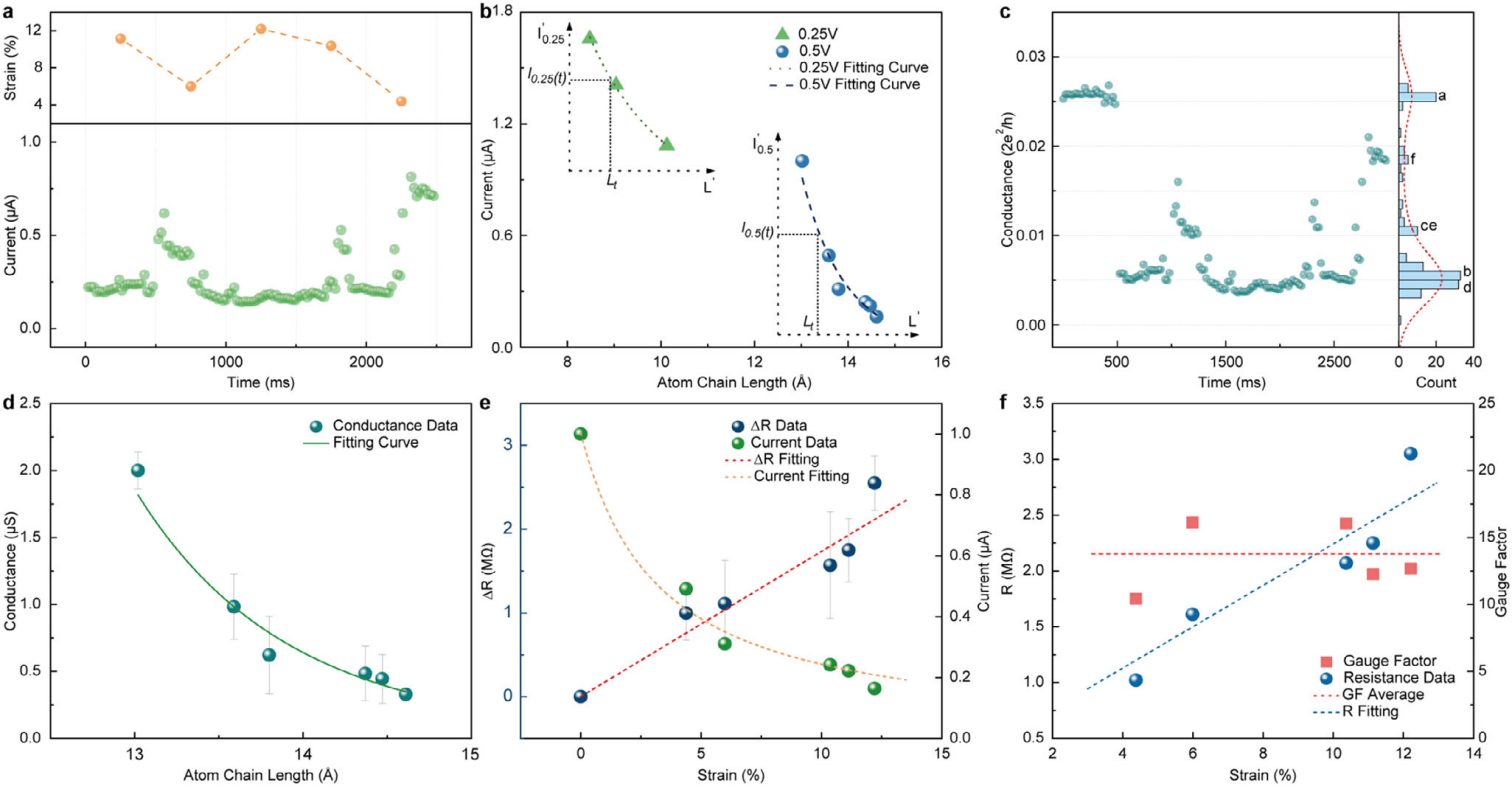
a) Changes in the current and strain of the atom chain over time. b) Relationship between chain current and atom chain length at different bias voltages. c) Conductance and histogram of conductance values in series time. d) The relationship curve between atom chain conductance and length. e) The values of ΔR currents at different strains and their corresponding fitting curves. ΔR exhibits a good linear relationship, while the current curve follows an inverse proportion function. f) The changes in the resistance of the single‐atom chain under different strains and the piezoresistance coefficients at different strains with good consistency.

At room‐temperature, after aligning the Ag crystal planes on both sides of the chain in the *z*‐direction using the nanomanipulator, a fixed bias voltage is applied at both ends of the single‐atom chain. As shown in **Figure** **3**a, during the atom chain tensioning process over 2500 ms with a 0.5 V bias voltage, corresponding to Figure 1b–f, the strain in the atom chain decreases from 11.1% to 5.9% at 500 ms, with a corresponding noticeable increase in current. In the subsequent 1000 ms, the strain increases to 12.2%, nearly returning to the initial strain level. At this point, the current decreases to a level slightly lower than the initial value, ≈80% of the original current. Between 1500 and 2000 ms, the strain in the single‐atom chain decreases sharply, and the current concurrently drops. During the 2000 to 2500 ms period, the atom chain is compressed again, the strain decreases further, and the current passing through the chain rises synchronously.

The dynamic processes of nanostructures (such as quantum dots, crystal interfaces, and low‐dimensional materials) occur on extremely short timescales, often in the microsecond range or even smaller. These rapid changes in crystal structure or spatial configuration would be missed if solely relying on HRTEM imaging. To address this limitation, additional strain sensing units with high temporal and spatial resolution are employed during in situ high‐resolution characterization. These strain sensors not only match the spatial resolution of TEM but also effectively compensate for the low imaging frequency of TEM.

In this study, we utilize a 1D single‐atom chain to make direct physical contact with the nanostructure under investigation. Due to the stable bonding between the single‐atom chain and the nanostructure, which occurs under the combined influence of an external electric field and van der Waals forces, the bonding remains intact within a certain range of external stresses. By measuring the current passing through the single‐atom chain, we can indirectly obtain the lattice‐level strain in the contact region of the structure being studied. This method doesn't require complex electrical and mechanical equipment and only necessitates high‐precision electrical testing equipment with a high sampling frequency, enabling in situ electrical and high‐resolution characterization of nanostructures in the TEM environment.

Two different regions of Ag crystal structures were selected, and fixed bias voltages of 0.5 and 0.25 V were applied to the two respective regions. The nanomanipulator was then moved in sub‐nanometer steps to simulate the lattice‐level motion of the crystal structure. During this process, the current passing through the single‐atom chain was measured. As shown in Figure 3b, the relationship between atomic chain length and current during lattice changes was obtained. The data distribution and fitting results indicate that the chain length and current follow a well‐established inverse‐proportional relationship. Although a smaller bias voltage was applied, the shorter length of the atom chain resulted in a smaller chain conductance and a higher current through the single‐atom chain. In an ideal contact scenario, for chains of equal length, the current should be proportional to the applied bias voltage, i.e., *I_0.5_* = 2*I_0.25_*. However, due to the different crystal regions selected for measurement and varying contact resistances, the shape of the inverse proportional curves for chain length and current was altered, and the position of these curves was shifted.

The experimentally obtained electrical conductance values were compared to the theoretical quantum conductance (*G_0_* ≈ 77.48 µS), which represents the ideal conductance for a single‐atom contact.^[^ ^36^, ^37^, ^38^ ^]^ In experiments, the conductance is influenced by a range of factors, including the characterization temperature, interatomic spacing, the number of atoms in the contact region, and the specific material properties, all of which can significantly affect the overall electrical behavior.^[^ ^39^ ^]^ Figure 3c above shows the conductance and the corresponding occurrence frequency distribution for the six atom chains from Figure 1a–f. The chain lengths vary from 11.5 to 15 Å, with conductance values ranging from 0.004*G_0_* to 0.028*G_0_*, indicating discrete variations. The histogram on the right displays the frequency of each corresponding conductance value. An obvious peak is observed ≈0.026*G_0_*, representing the conductance value at the moment Figure 1a and lasting for 0.5 s. The relationship between chain length and conductance is shown in Figure 3d for an applied bias voltage of 0.5 V. The chain length and current follow a relatively good inverse proportionality relationship. The experimentally measured conductance of the single‐atom chain is lower than theoretical quantum conductance, a phenomenon attributed to the fact that the in situ electrical characterization of the single‐atom chain was conducted at room‐temperature rather than in an ultralow temperature environment.

In situ high resolution and electrical characterizations demonstrate that the Ag single‐atom chain exhibits a significant piezoresistance effect. This also highlights the potential application of the piezoresistance Ag single‐atom chain as an in situ lattice‐level displacement sensor in the TEM environment. For the single‐atom chain sensor, within the strain range where the chain structure remains stable, strain can be approximated as the sole variable affecting the resistance. With a fixed number of 5 Ag atoms, the maximum tensioning strain reaches *ε* = 12.2% under a 0.5 V bias, and the corresponding current decreases in a typical inverse proportional trend as strain increases. Meanwhile, the resistance increment is directly proportional to the strain, with a resistance increase of 185 kΩ for every 1% strain, showing good linearity. This indicates that the resistance change in the Ag single‐atom chain during the strain process follows the metal piezoresistance theory, indirectly confirming the stability and reliability of the piezoresistance sensing.

The gauge factor (GF) for the metal piezoresistance effect is defined as the rate of change in resistance per unit strain and is influenced by the material, dimensions, and structure of the metal.^[^ ^40^, ^41^ ^]^ As shown in Figure 3f, the single‐atom chain exhibits a positive piezoresistance effect similar to that of macroscopic Ag wires, and the gauge factor remains approximately constant. The resistance of the atom chain maintains a good linear relationship with strain. The longitudinal intercept of the resistance is related to the number of atoms, the initial length of the chain, and the contact resistance in the measurement area. The piezoresistance effect varies significantly between macroscopic and microscopic scales. In semiconductor materials, the scale effect of piezoresistance is more pronounced, while the effect is relatively weaker in metallic materials. At the macroscopic scale, the piezoresistance coefficient of Ag wires is 3.35 ± 0.13, but the piezoresistance coefficient of the Ag single‐atom chain at room‐temperature on the nanoscale has an average value of 13.5 in this experiment, which is four times the macroscopic value, demonstrating the giant piezoresistance effect of metals at room‐temperature.

Based on the previous discussion, the Ag single‐atom chain exhibits a significant positive piezoresistance effect within the strain range where its structure remains stable, and the piezoresistance coefficient remains relatively constant. As a strain sensing structure, it is not limited by the TEM platform system and only depends on the sampling frequency and accuracy of the external current measurement equipment. This provides a strong resistance to interference, and the sensor offers extremely high temporal resolution, greatly increasing the likelihood of capturing key strains during dynamic changes in nanostructures. This capability serves as a significant complement to current in situ characterization techniques based on TEM.

## 4 Simulation of Strain and Electron Transport Processes in Ag Single‐Atom Chains Based on Density Functional Theory

The piezoresistance effect exhibited by single‐atom metal nanowires holds significant potential for the characterization of nanomaterials and devices. This study demonstrates that Ag atom chains have a high piezoresistance coefficient (4–5 times greater than the bulk value) in a vacuum environment at room‐temperature with highly stable. The impact of atomic‐level strain on the transport of charge carriers within these chains remains poorly understood, which may be key to fully elucidating the mechanisms underlying atomic‐scale piezoresistance phenomena.^[^ ^42^ ^]^ Therefore, this paper uses DFT simulations to investigate the internal electronic transport process of single‐atom chains under different strain conditions.

This study presents DFT calculations on the conductivity of Ag single‐atom chains under various strain conditions, revealing excellent agreement between theoretical predictions and experimental results. The physical model comprises an Ag atom chain connected to two semi‐infinite Ag(100) metal electrodes, structured into three distinct regions: the left electrode, the right electrode, and the central extended scattering region, which contains the Ag single‐atom chain. The left and right electrodes are treated as perfect crystals, while the central scattering region contains a five‐atom Ag chain, with two atoms at each end in contact with the electrodes. The electrode is considered a perfect bulk material. We used the density‐functional‐based tight‐binding (DFTB) approach, implemented via the DFTB+ module, to perform electronic structure calculations.^[^ ^43^, ^44^, ^45^ ^]^

**Figure 4:**
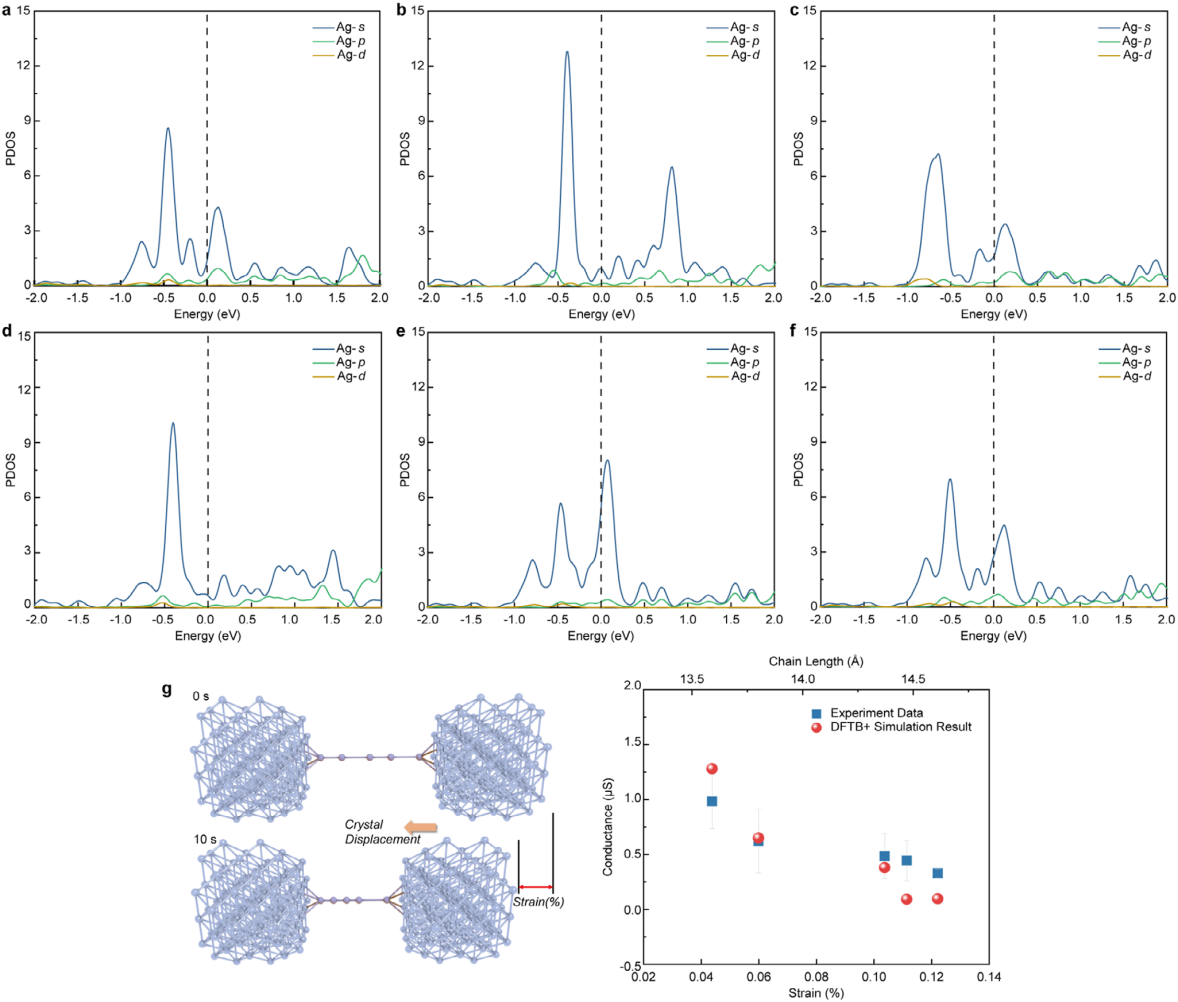
a–f) The PDOS of each Ag single‐atom chain at equilibrium position under 0.5 V bias voltage. g) Comparison between the single‐atom chain conductance values obtained from DFTB+ simulations and those measured in experiments.

We calculated the projected density of states (PDOS) for six sets of Ag single‐atom chains at equilibrium positions under applied stress, corresponding to the six keyframes in Figure 1. The results, shown in **Figure** **4**a–f, indicate that the primary transport channel in Ag atoms is formed by *s‐*orbital electrons, with minimal contributions from *p‐* and *d‐*orbital electrons. The PDOS spectrum near the Fermi level is similar for strains less than 10% compared to the unstrained state. As strain increases, peak heights far from the Fermi level show significant variation. When the strain exceeds 10%, the PDOS spectrum shape diverges notably from the unstrained case, and the band structure gradually develops a bandgap near the Fermi level. A sudden shift occurs ≈10% strain; beyond this point, with slight further strain (1–2%), the spectrum remains stable below the Fermi level. This behavior suggests that strain influences the strength of metallic bonds between atoms. Small strains cause a slight splitting at the valence band edge, leaving conductivity near the Fermi level unaffected. At higher strains, however, conductivity near the Fermi level is significantly impacted. Nevertheless, the overall electrical conductivity of the Ag atom chain remains fundamentally stable, demonstrating that the atom chain retains favorable mechanical properties and conductivity within a specific strain range.

The comparison between the simulated electrical conductivity of the Ag single‐atom chain, calculated using DFTB+, and experimentally measured conductivity is shown in Figure 4g. The simulation results align closely with in situ electrical characterization data obtained from TEM, demonstrating the physical validity of the simulation. This agreement also indirectly supports the theoretical explanation of the effect of strain on conductivity obtained from the simulation. Moreover, these findings confirm that Ag single‐atom chains exhibit robust mechanical stability and pronounced positive piezoresistance effects at room‐temperature, highlighting their potential for in situ lattice‐level sensing and electrical characterization.

## 5 Conclusion

In this study, we fabricated structurally stable Ag single‐atom chains by in situ tensioning of Ag nanocrystals under a Cs‐TEM environment. The dynamic structure of these chains was subjected to in situ high‐resolution strain and electrical characterizations. We investigated the lattice‐level piezoresistance effects under different atomic spacings and strains, which exhibited significant piezoresistance behavior at room‐temperature. With DFT simulations, we elucidated the mechanism of atom chain piezoresistive behavior, providing both experimental and theoretical insights into the piezoresistance at the atomic scale. By combining the atomic‐level piezoresistance of single‐atom chains with in situ high sampling rate measurements, the single‐atom chain is proposed as a lattice‐level dynamic strain sensing functional structure. The strain‐sensitive atomic chain/nanowire structures can effectively compensate for the low sampling rate issue associated with vision‐based sensing in micro‐ and nanoscale characterization. This study conducted at the minimal scale within classical theoretical, provides both experimental and theoretical foundations for the future application of nanowire structures in micro‐ and nanoscale strain sensors. It broadens the theoretical support for the piezoresistive effect in nanoscale strain sensors, further confirming its significant application potential and offering new insights into in situ strain and electrical characterization of nanomaterials and structures.

## 6 Experimental Section

### Sample Preparation and In Situ Nanofabrication of Single Ag Atom Chain

The semi‐copper (Cu) grid was used to pick up a small amount of Ag nanosheets, while the W needle tip was also coated with Ag nanosheets. The semi‐Cu grid was placed on the sample stage of the in situ electrical TEM single tilt holder (FE02‐DT, PicoFemto). The sample was cleaned using plasma before insertion. The high‐resolution Cs‐TEM (Themis Z, Thermo Fisher Scientific) at 300 kV. A fixed bias voltage was applied to the sample using the PicoFemto controller, with the semi‐Cu grid as the negative electrode and the W needle tip as the positive electrode. Based on the contrast differences in TEM mode, areas of the thin Ag nanosheets at the W needle tip with lower contrast and stable structure were selected to gradually contact similar areas on the semi‐Cu grid side. A fixed bias (0.1, 0.25, 0.5, 1 V) was applied when the distance between the two poles was less than 10 nm. When the edges of the two crystals contacted, crystal interface fusion occurred, similar to the cold welding of crystal planes. When two crystal faces separate, the silver atoms originally belonging to the two faces recombine through metallic bonds to form a 1D atomic chain.

### Chain Current Measurement

In the TEM sample holder, the semi‐Cu grid end served as the negative electrode, and the W (tungsten) needle end acted as the positive electrode. A fixed bias voltage was applied across these two ends to measure the contact current and the atom chain current. The positive and negative electrodes were connected to the external coaxial cable through wires inside the sample rod, passing through a vacuum device at the tail of the rod. For the control and output of the bias voltage, the PicoFemto Holder controller was employed, along with a KEITHLEY SourceMeter (Model 2634b). The coaxial cable was connected to V‐out and Sample for voltage output, and to I‐in and Tip for current input. The standard voltage mode allowed for an output voltage of up to ±10 V. In this experiment, DC voltage was used with a sampling rate of 50 Hz.

### DFT Simulation

Lattice vibrations and electronic transport across the Ag atoms were described using Slater–Koster parameters. The electronic configuration of Ag atoms (4d¹⁰5s¹) was represented using the HYB library. Nonperiodic boundary conditions were applied along the transport direction of the single‐atom chain, while periodic boundary conditions were employed for the two perpendicular directions, simulating infinite length. The system was maintained at 300 K to match experimental conditions. A reciprocal space grid with a 0.02 Å⁻¹ spacing was utilized, and total energy convergence was set to 10⁻⁸ Hartree. The transmission spectrum was calculated using nonequilibrium Green's functions. Charge calculations were performed self‐consistently, with a convergence tolerance of 1 × 10^−8^. The Brillouin zone was sampled using the Monkhorst–Pack method with a resolution finer than 0.02 Å⁻¹.

## Conflict of Interest

The authors declare no conflict of interest.

## Supporting information



Supporting Information

Supplemental Video 1

Supplemental Video 2

Supplemental Video 3

## Data Availability

The data that support the findings of this study are available from the corresponding author upon reasonable request.

## References

[1] A. Sobhani , M. W. Knight , Y. Wang , B. Zheng , N. S. King , L. V. Brown , Z. Fang , P. Nordlander , N. J. Halas , Nat. Commun. 2013, 4, 1643. 23535664 10.1038/ncomms2642

[2] J. Yang , W.‐H. Li , H.‐T. Tang , Y.‐M. Pan , D. Wang , Y. Li , Nature 2023, 617, 519. 37198309 10.1038/s41586-023-05886-z

[3] S. Seo , S. H. Jo , S. Kim , J. Shim , S. Oh , J. H. Kim , K. Heo , J. W. Choi , C. Choi , S. Oh , D. Kuzum , H. S. P. Wong , J. H. Park , Nat. Commun. 2018, 9, 5106. 30504804 10.1038/s41467-018-07572-5 PMC6269540

[4] W. Q. Zhu , R. Esteban , A. G. Borisov , J. J. Baumberg , P. Nordlander , H. J. Lezec , J. Aizpurua , K. B. Crozier , Nat. Commun. 2016, 7, 11495. 27255556 10.1038/ncomms11495 PMC4895716

[5] J. Teeter , N. Y. Kim , T. Debnath , N. Sesing , T. Geremew , D. Wright , M. Chi , A. Z. Stieg , J. Miao , R. K. Lake , T. Salguero , A. A. Balandin , Adv. Mater. 2024, 36, 2409898. 10.1002/adma.202409898 39400439

[6] S. L. Wang , Z. W. Shan , H. Huang , Adv. Sci. 2017, 4, 1600332. 10.1002/advs.201600332 PMC5396167 28435775

[7] T. Xin , Y. Zhao , R. Mahjoub , J. Jiang , A. Yadav , K. Nomoto , R. Niu , S. Tang , F. Ji , Z. Quadir , D. Miskovic , J. Daniels , W. Xu , X. Liao , L.‐Q. Chen , K. Hagihara , X. Li , S. Ringer , M. Ferry , Sci. Adv. 2021, 7, abf3039. 10.1126/sciadv.abf3039 PMC8172136 34078600

[8] M. Höfling , X. Zhou , L. M. Riemer , E. Bruder , B. Liu , L. Zhou , P. B. Groszewicz , F. Zhuo , B.‐X. Xu , K. Durst , X. Tan , D. Damjanovic , J. Koruza , J. Rödel , Science 2021, 372, 961. 34045350 10.1126/science.abe3810

[9] J. Z. Cai , C. Griesbach , S. G. Ahnen , R. Thevamaran , Acta Mater. 2023, 249, 118807.

[10] E. Gil‐Santos , D. Ramos , J. Martínez , M. Fernández‐Regúlez , R. García , A. San Paulo , M. Calleja , J. Tamayo , Nat. Nanotechnol. 2010, 5, 641. 20693990 10.1038/nnano.2010.151

[11] S. R. C. Vivekchand , U. Ramamurty , C. N. R. Rao , Nanotechnology 2006, 17, S344.

[12] R. Li , J. Zhao , B. Liu , D. Wang , Adv. Mater. 2024, 36, 2308653. 10.1002/adma.202308653 37779465

[13] T. T. Cui , L. X. Li , C. L. Ye , X. Y. Li , C. X. Liu , S. H. Zhu , W. Chen , D. S. Wang , Adv. Funct. Mater. 2022, 32, 2108381.

[14] S. Ning , H. Ou , Y. Li , C. Lv , S. Wang , D. Wang , J. Ye , Angew. Chem., Int. Ed. 2023, 62, 202302253. 10.1002/anie.202302253 37012479

[15] N. Wang , P. F. Ou , R. K. Miao , Y. X. Chang , Z. Y. Wang , S. F. Hung , J. Abed , A. Ozden , H. Y. Chen , H. L. Wu , J. E. Huang , D. J. Zhou , W. Y. Ni , L. Z. Fan , Y. Yan , T. Peng , D. Sinton , Y. C. Liu , H. Y. Liang , E. H. Sargent , J. Am. Chem. Soc. 2023, 145, 7829. 37010254 10.1021/jacs.2c12431

[16] N. Daelman , M. Capdevila‐Cortada , N. López , Nat. Mater. 2019, 18, 1215. 31384029 10.1038/s41563-019-0444-y

[17] M. Pozzo , D. Alfè , P. Lacovig , P. Hofmann , S. Lizzit , A. Baraldi , Phys. Rev. Lett. 2011, 106, 135501. 21517393 10.1103/PhysRevLett.106.135501

[18] N. Yodsin , C. Rungnim , V. Promarak , S. Namuangruk , N. Kungwan , R. Rattanawan , S. Jungsuttiwong , Phys. Chem. Chem. Phys. 2018, 20, 21194. 30083668 10.1039/c8cp02976h

[19] T. Ding , X. Liu , Z. Tao , T. Liu , T. Chen , W. Zhang , X. Shen , D. Liu , S. Wang , B. Pang , D. Wu , L. Cao , L. Wang , T. Liu , Y. Li , H. Sheng , M. Zhu , T. Yao , J. Am. Chem. Soc. 2021, 143, 11317. 34293258 10.1021/jacs.1c05754

[20] S. Tian , Q. Fu , W. Chen , Q. Feng , Z. Chen , J. Zhang , W.‐C. Cheong , R. Yu , L. Gu , J. Dong , J. Luo , C. Chen , Q. Peng , C. Draxl , D. Wang , Y. Li , Nat. Commun. 2018, 9, 2353. 29907774 10.1038/s41467-018-04845-x PMC6003949

[21] Z. Li , X. Guo , Y. Jin , F. Andreoli , A. Bilgin , D. D. Awschalom , N. Delegan , F. J. Heremans , D. Chang , G. Galli , A. A. High , Nat. Photonics 2024, 18, 1113.

[22] T. H. Taminiau , F. D. Stefani , F. B. Segerink , N. F. van Hulst , Nat. Photonics 2008, 2, 234.

[23] G. Wolfowicz , C. P. Anderson , A. L. Yeats , S. J. Whiteley , J. Niklas , O. G. Poluektov , F. J. Heremans , D. D. Awschalom , Nat. Commun. 2017, 8, 1876. 29192288 10.1038/s41467-017-01993-4 PMC5709515

[24] T. Kizuka , K. Monna , Phys. Rev. B 2009, 80, 205406.

[25] T. Kizuka , Phys. Rev. B 2008, 77, 155401.

[26] N. D. Lang , Phys. Rev. B 1995, 52, 5335. 10.1103/physrevb.52.5335 9981724

[27] C. J. Muller , J. M. van Ruitenbeek , L. J. de Jongh , Phys. Rev. Lett. 1992, 69, 140. 10046209 10.1103/PhysRevLett.69.140

[28] N. D. Lang , Phys. Rev. Lett. 1997, 79, 1357.

[29] A. A. Balandin , F. Kargar , T. T. Salguero , R. K. Lake , Mater. Today 2022, 55, 74.

[30] L. Cui , W. Jeong , S. Hur , M. Matt , J. C. Klöckner , F. Pauly , P. Nielaba , J. C. Cuevas , E. Meyhofer , P. Reddy , Science 2017, 355, 1192. 28209640 10.1126/science.aam6622

[31] A. I. Yanson , G. R. Bollinger , H. E. van den Brom , N. Agraït , J. M. van Ruitenbeek , Nature 1998, 395, 783.

[32] J. M. Wen , S. L. Chang , J. W. Burnett , J. W. Evans , P. A. Thiel , Phys. Rev. Lett. 1994, 73, 2591. 10057099 10.1103/PhysRevLett.73.2591

[33] C. L. Liu , J. M. Cohen , J. B. Adams , A. F. Voter , Surf. Sci. 1991, 253, 334.

[34] Y. Asai , H. Fukuyama , Phys. Rev. B 2005, 72, 085431.

[35] F.‐T. Liu , Y. Cheng , F.‐B. Yang , X.‐R. Chen , Phys. E: Low‐Dimens. Syst. Nanostruct. 2014, 56, 96.

[36] N. Agraït , J. G. Rodrigo , S. Vieira , Phys. Rev. B 1993, 47, 12345. 10.1103/physrevb.47.12345 10005423

[37] J. M. Krans , J. M. van Ruitenbeek , V. V. Fisun , I. K. Yanson , L. J. de Jongh , Nature 1995, 375, 767.

[38] H. Ohnishi , Y. Kondo , K. Takayanagi , Nature 1998, 395, 780.

[39] E. Scheer , N. Agraït , J. C. Cuevas , A. L. Yeyati , B. Ludoph , A. Martín‐Rodero , G. R. Bollinger , J. M. van Ruitenbeek , C. Urbina , Nature 1998, 394, 154.

[40] A. S. Fiorillo , C. D. Critello , S. A. Pullano , Sens. Actuators A: Phys. 2018, 281, 156.

[41] R. He , P. Yang , Nat. Nanotechnol. 2006, 1, 42. 18654140 10.1038/nnano.2006.53

[42] J. X. Cao , X. G. Gong , R. Q. Wu , Phys. Rev. B 2007, 75, 233302.

[43] J. Kullgren , M. J. Wolf , K. Hermansson , C. Köhler , B. Aradi , T. Frauenheim , P. Broqvist , J. Phys. Chem. C 2017, 121, 4593.

[44] G. Dolgonos , B. Aradi , N. H. Moreira , T. Frauenheim , J. Chem. Theory Comput. 2010, 6, 266. 26614337 10.1021/ct900422c

[45] B. Szűcs , Z. Hajnal , R. Scholz , S. Sanna , T. Frauenheim , Appl. Surf. Sci. 2004, 234, 173.

