# Supplementary material for: Atomic‐Level Strain Sensing and Piezoresistance Effect in a 1D Single‐Atom Chain
Source: Adv Sci (Weinh). 2025 Apr 26;12(23):2500553. doi: 10.1002/advs.202500553 (PMC12199423; doi:10.1002/advs.202500553)
Supplement: Supplementary file 1 — Supporting Information [file ADVS-12-2500553-s004.docx]

Supporting Information

**Atomic-Level Strain Sensing and Piezoresistance Effect in a One-Dimensional Single Atom Chain**

*Zhi Qu, Wenqi Zhang, Shuideng Wang, Donglei Chen, Yiqing Yao, Mingxing Cheng and Lixin Dong**

**Detail list of supplementary figures**

Figure S1: Schematic Diagram of an In-Situ Single Tilt Electrical Holder and Double Aberration-Corrected Transmission Electron Microscope.

Figure S2: Stretching Process of Ag Atomic Chains - Part 1

Figure S3: Stretching Process of Ag Atomic Chains - Part 2.

Figure S4: Changes in Current During the Peeling Process of Atomic Chains.

Figure S5: Electrical Characterization of Single and Double Row Atomic Chains.

Figure S6. Histogram of Conductance Values Measured and Counted in Deformation Process.

Figure S7. High-resolution Characterization Images of Silver Atom Chain Crystal Planes on Both Sides and In-situ Characterization of Tunnel Junction Current.


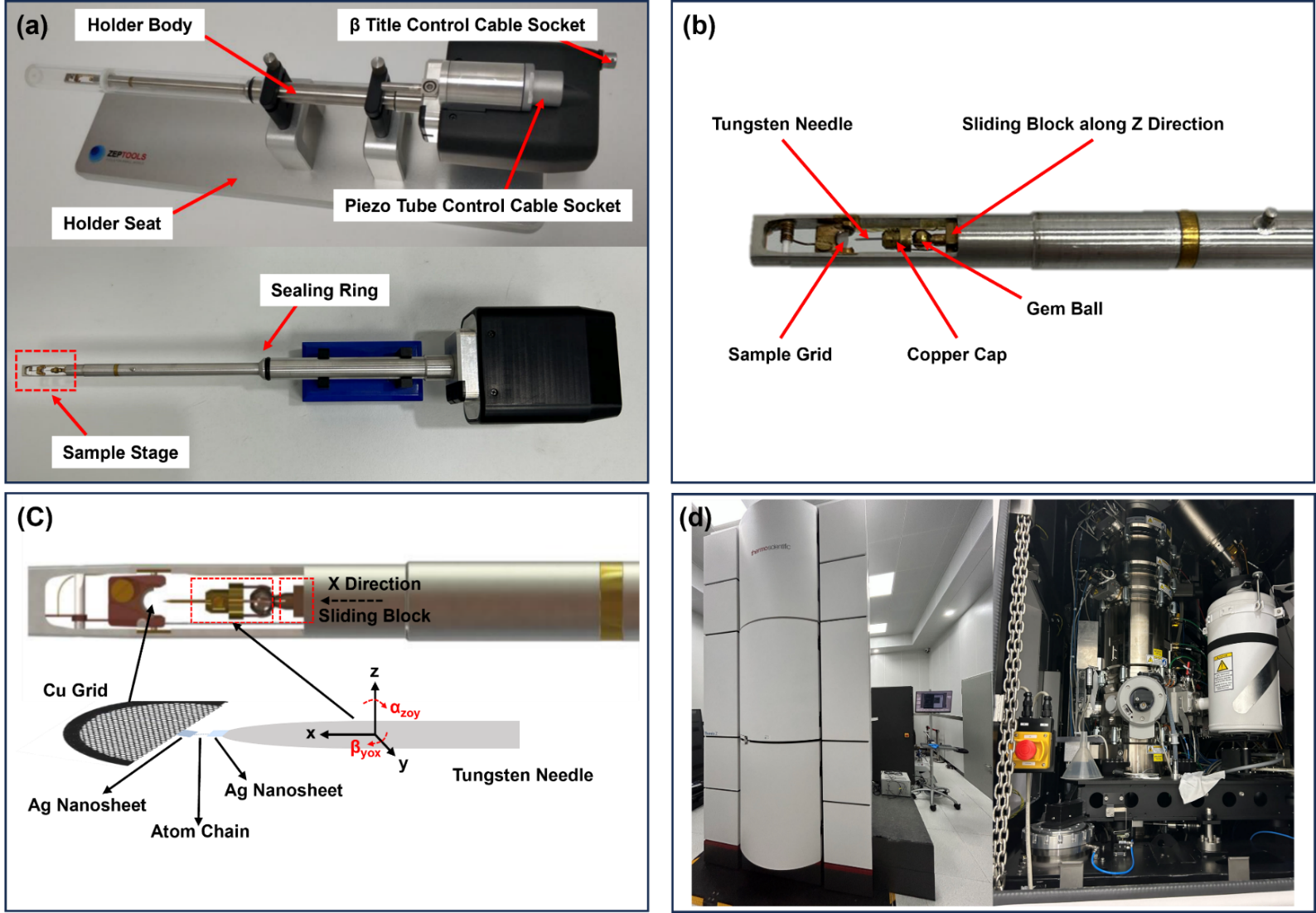


**Figure S1.** Schematic diagram of an in-situ single tilt electrical holder and double aberration-corrected transmission electron microscope. a-c) the design and functional components of in-situ single tilt electrical holder. The in-situ holder, composed of a piezoelectric nanomanipulator consisting of a Sliding block, a gem ball, and a copper cap, enables the control of the tungsten needle tip with three degrees of freedom. External voltage signals can be directed into the sample stage via a coaxial cable through the control cable socket. d) FEI Themis Z TEM with double corrected, resolution 60pm.


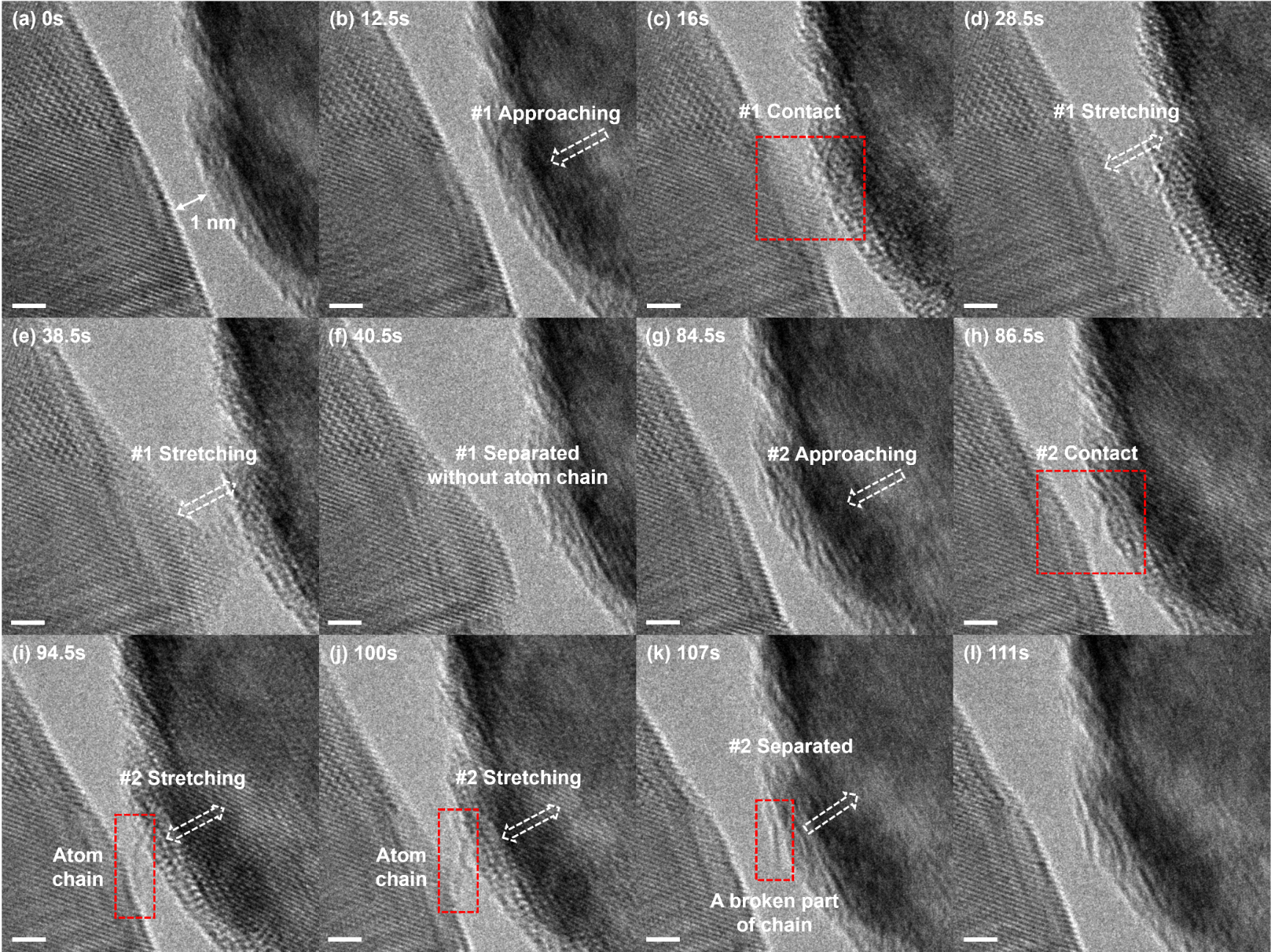


**Figure S2.** Stretching process of Ag atomic chains - Part 1. In the images, a scale length of 1 nm is used, and a constant 1 V DC bias voltage is applied throughout the process. The sampling frequency for high-resolution in-situ TEM images is set at 500 ms per frame. a) The Ag nanosheet at the tip of the W needle, serving as the anode, gradually approaches the Ag nanosheets at the cathode, with a distance of 1 nm between them. b-c) The Ag nanosheet on the anode side (right) slowly moves closer to the cathode until they make their first contact. When the Ag crystal planes of the anode and cathode come into contact, atoms on the crystal planes rebond, combining in a way similar to cold welding. d-f) The piezoelectric nanomanipulator moves to the right, stretching the newly bonded crystal region, until it eventually breaks. No atomic chains are formed during this stretching process. g-h) Afterward, the anode on the right side is gradually brought closer to the cathode again, leading to a second contact. i) Once the contacted crystal regions have rearranged their lattice, the crystal area is stretched again. j) As the crystal planes of the anode and cathode are about to separate, atomic chains are peeled off from the surface of the crystal plane. k-l) With the continuation of the stretching, the atomic chains break.


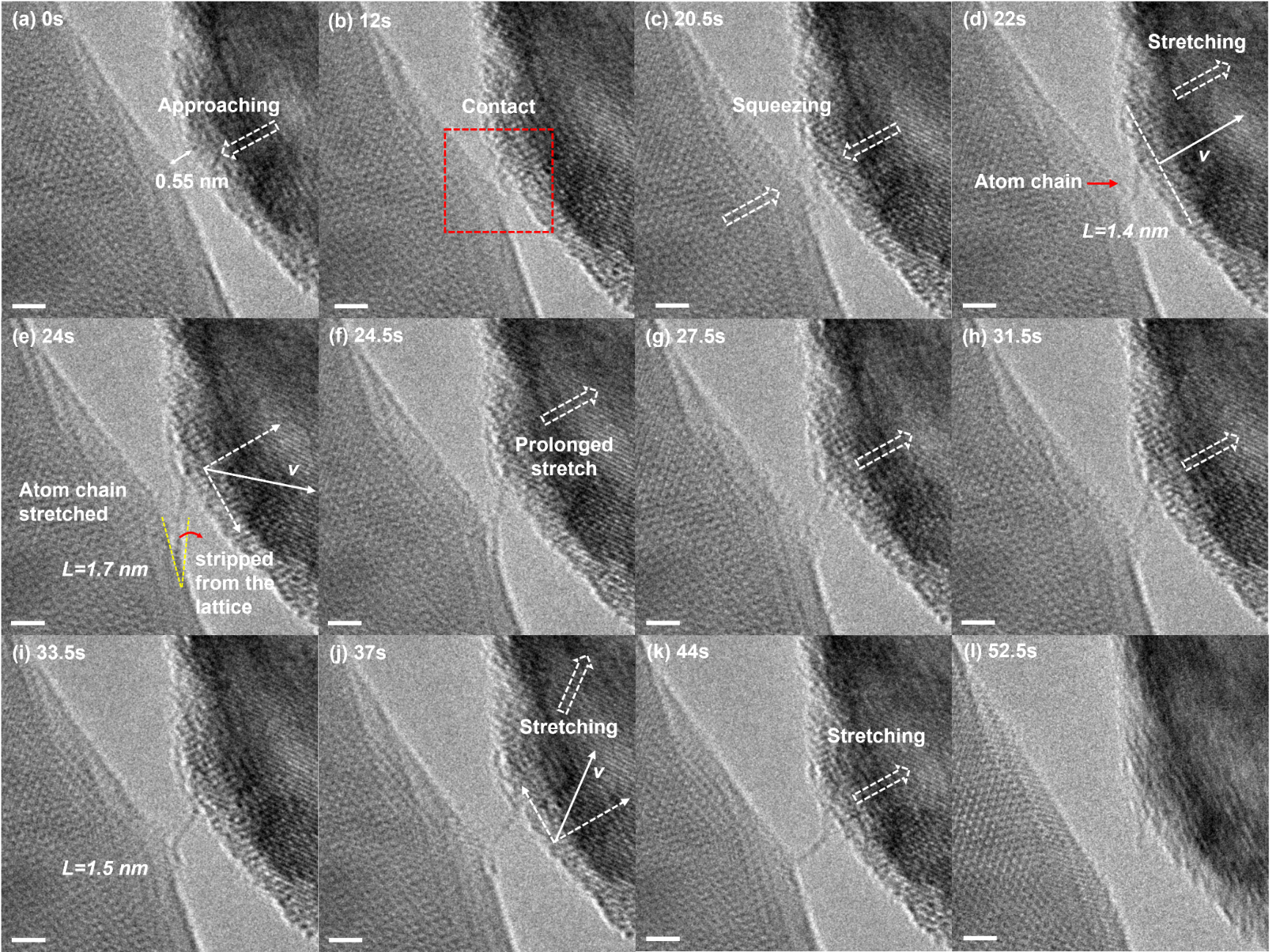


**Figure S3.** Stretching process of Ag atomic chains - Part 2. In the images, the scale length is set at 1 nm, and a constant 0.5 V DC bias voltage is applied throughout the process. The sampling frequency for high-resolution TEM images is maintained at 500 ms per frame. a, b) The nanomanipulator is controlled to gradually bring the right-side anode Ag nanosheet crystal closer to the cathode until they contact. c) A certain force is continuously applied, and after maintaining this state for a while, the manipulator is controlled to move in the direction away from the cathode. d) During the separation of the grain boundary, atomic chains peel off from the edge of the cathode. e-k) The crystal on the right side is then controlled to move along the base of the atomic chains in the direction away from the cathode, gradually peeling off longer atoms. Throughout the stretching process, the atomic chains maintain structural stability for a long time and exhibit excellent electrical properties. l) The stretching continues until the atomic chains break.


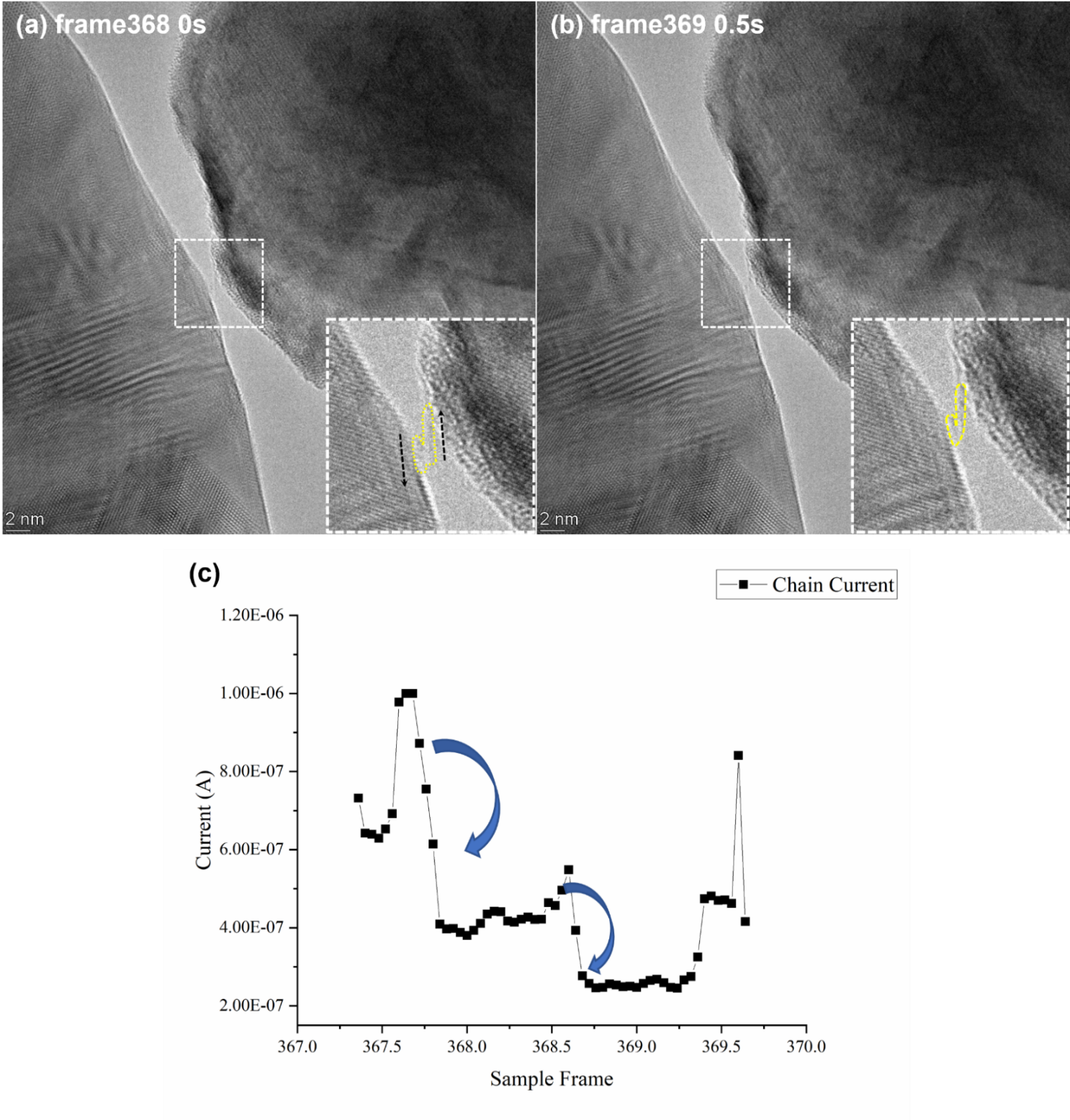


**Figure S4.** Changes in current during the peeling process of atomic chains. a-b) During the application of a bias voltage of 1 V, distinct changes in current are observed in response to the stretching, lateral movement, and necking of the atomic chains. c) These modifications in the atomic chain configuration result in noticeable step-like variations in the electrical current. This phenomenon can be attributed to the discrete changes in the electronic structure and conductive pathways as the atomic chain undergoes physical transformations. Each alteration in the atomic arrangement leads to a corresponding adjustment in the current flow, which is detectable as distinct steps or stages in the current's intensity or pattern.


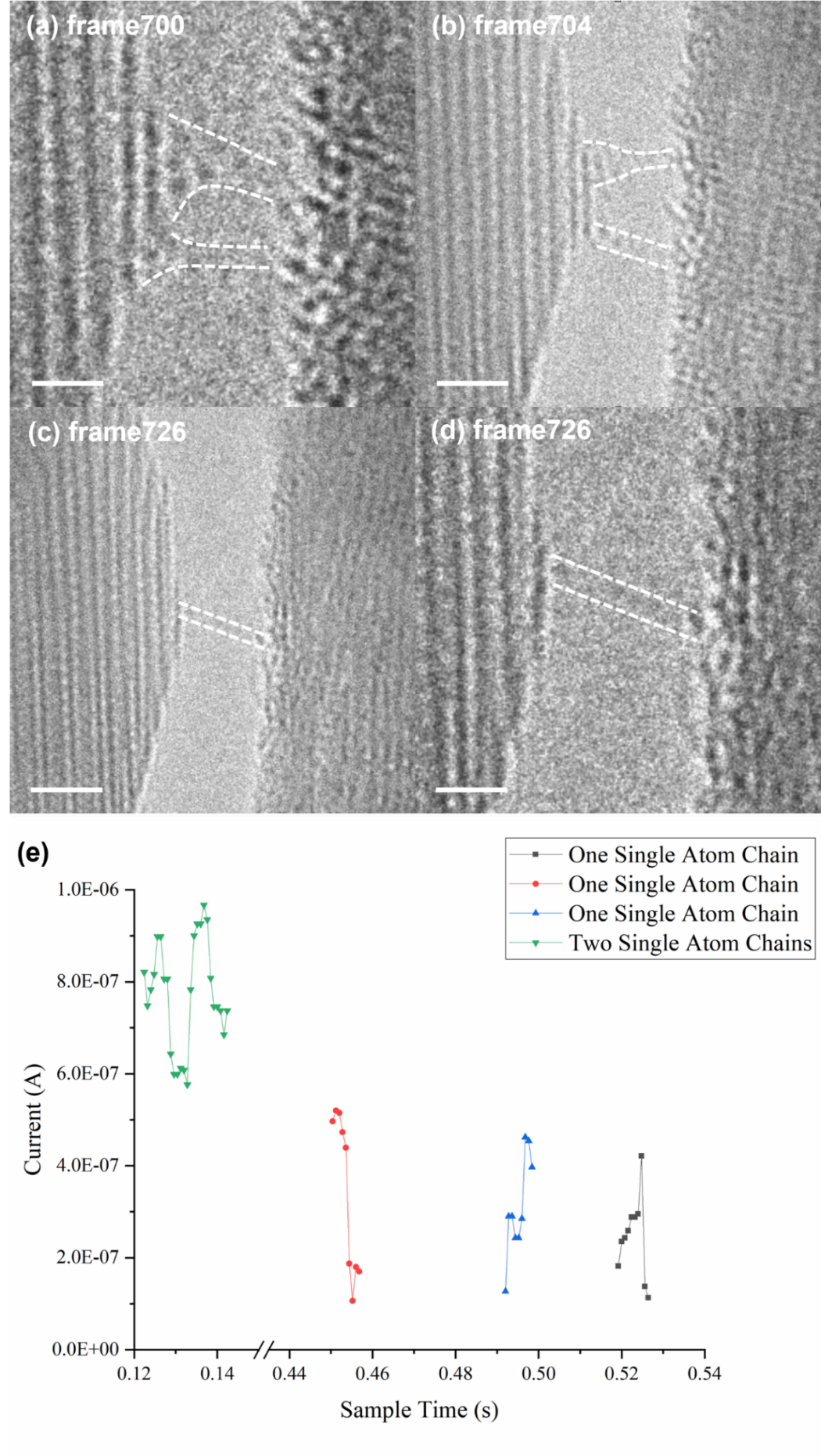


**Figure S5.** Electrical characterization of single and double row atomic chains. The bias voltage was fixed at 0.25 V. a-d) During the process of crystal stretching and grain boundary separation, single atomic chains and two parallel atomic chains were observed. e) Analysis of these two scenarios revealed that the current in the parallel atomic chains is approximately twice that of a single atomic chain.


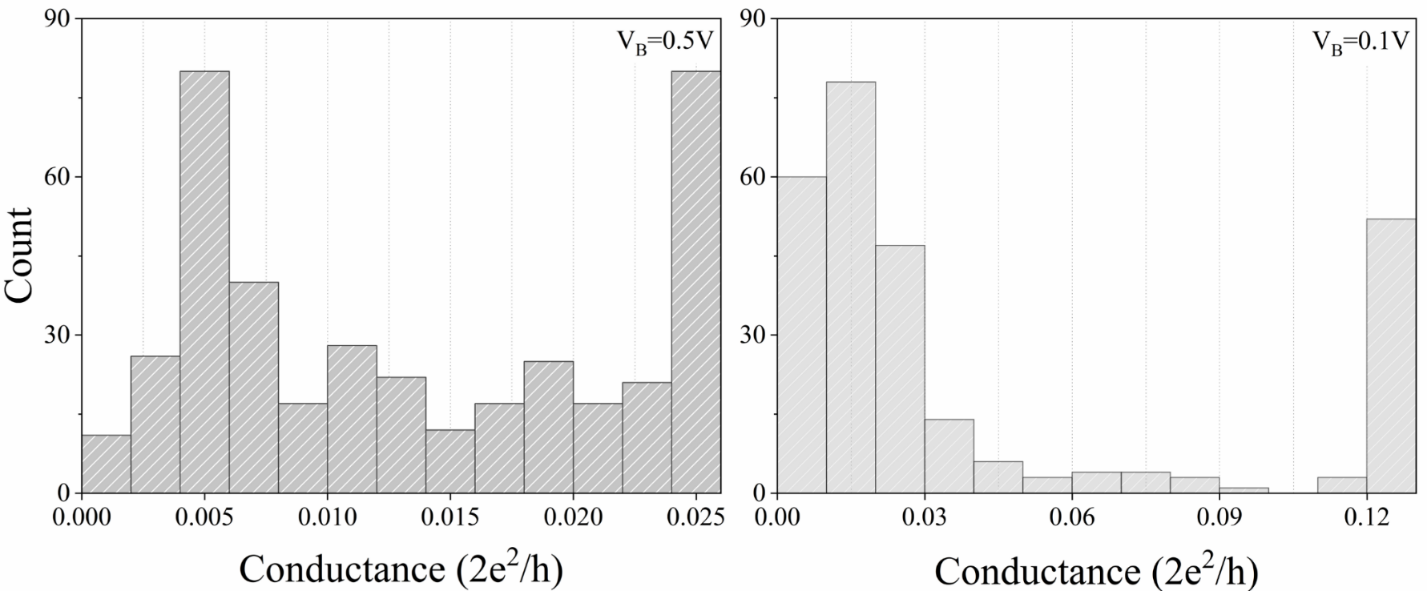


**Figure S6.** Histogram of conductance values measured and counted during deformation process. Voltage biased by 0.5 V and 0.1 V between electrodes.

**The Detailed Explanation and Calculation Process of Equivalent Capacitance**

The impact of nanoscale capacitors on the electron transport process in one-dimensional atomic chains can be analyzed from the following three aspects: (1) The capacitance value of the nanoscale capacitor formed between the two electrodes; (2) The charge accumulation and local electric field effects induced by the capacitance; (3) The Coulomb blockade effect caused by the capacitance. Below, a detailed explanation of how these three factors influence the electrical properties of the single-atom chain in our study is provided:

(1) Explanation from the perspective of the equivalent capacitance between the silver atom chain and the crystal planes on both sides.

In the absence of the atom chain, the entire system is considered a nanoscale capacitor. The closest distance between the two silver crystal planes is approximately 1.2 nm (with the average length of the atom chain being about 1.36 nm). However, at this distance, the effective area of the two plates is quite small. As shown in **Figure S7(a)**, the projection length of the crystal planes in the focal plane is approximately 10 nm (even though the width of the silver crystal at the end actuator is only 40 nm). In the direction perpendicular to the focal plane, we take the length as 100 nm (the sample was thinned using FIB, controlled to be below 100 nm, so we take 100 nm here).


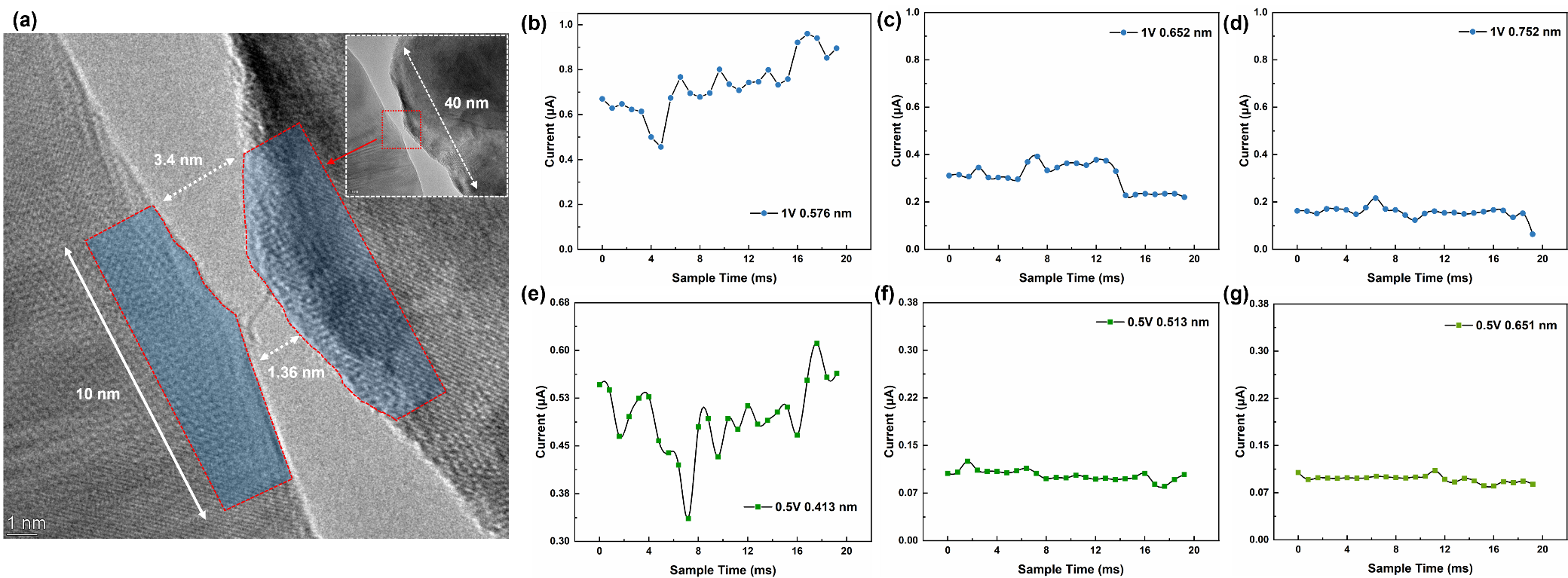


**Figure S7.** High-resolution characterization images of the silver atom chain's crystal planes on both sides and in-situ characterization of tunnel junction current. a) In this study, the width of the equivalent capacitance plates of the silver crystal regions at both ends of the atom chain is taken as a maximum of 40 nm, and the width of the entire silver crystal at the probe tip is also only 40 nm. b-g) Under the same experimental design, without the atom chain, these are measurements of tunnel current for different tunnel gaps in a single tunnel junction experiment, with fixed bias voltages of 1V and 0.5 V.

Here, we calculate the possible maximum capacitance value. In the case of the largest possible capacitance area (assuming the entire probe end actuator acts as the capacitor), the area is given by $A=40 nm\times100 nm=0.004 {\mu m}^{2}$. The spacing is uniformly taken as the minimum distance of 1.2 nm (in reality, only a very small region has a spacing of 1.2 nm. The region with a spacing of less than 3.5 nm accounts for about one-fourth of the total area, as shown in Figure S7(a)). Based on the classical capacitance formula, the maximum capacitance between the two crystal plates is approximately:

$C_{max}=\frac{\epsilon A}{d}\approx\frac{8.854\times{10}^{-12}\frac{F}{m}\times40\times{10}^{-9} m\times100\times{10}^{-9} m}{1.2\times{10}^{-9} m}=2.951\times{10}^{-17} F$ (1)

where $\epsilon=8.854\times{10}^{-12} F/m$ is the permittivity of vacuum, *A* is the plate area, and *d* is the plate separation.

Even with this calculation, the capacitance per unit area is $C_{A}=2.95 \mu F/\mathrm{cm}^{2}$. Although the distance between the two crystal planes is very small (1.2 nm), the total area of the crystal planes (equivalent capacitor plates) is also very limited. Therefore, the overall equivalent capacitance of the system is not particularly large, and correspondingly, its effect on the electron transport in the atom chain system is not very significant.

(2) Explanation from the perspective of charge accumulation and local electric field effects potentially inducing electron tunneling in the adjacent crystal (capacitor) structures.

In our separate in-situ high-resolution and electrical characterization experiments on tunnel junction tunneling current in TEM, the material, experimental setup, and operational methods are identical to those used in this study. Under fixed bias voltages of 0.1 V, 0.25 V, 0.5 V, and 1 V, noticeable tunneling currents were only observed when the tunnel junction gap was below 0.7 nm, as shown in Figure S7(b-g).

In contrast, this experiment applies 0.5 V and 0.25 V voltages, with an average atom chain length of 1.36 nm and an electrode spacing exceeding 1.2 nm. This indicates that in our atom chain system, tunneling effects induced by local charge accumulation and electric field effects have negligible influence on the measured chain current.

(3) Explanation from the perspective of Coulomb blockade effects.

Coulomb blockade refers to the phenomenon in nanoscale conductors (typically quantum dots, atom chains, etc.) where electron transport is hindered due to the discreteness of charge and capacitive effects. When an electron needs to overcome the energy required for charge redistribution, its transmission is impeded, resulting in a "blockade" or a significant reduction in current flow.

Here, we analyze the Coulomb blockade effect by calculating the Coulomb energy, which is defined as the energy barrier that must be overcome to add or remove a single electron.

$E_{C}=\frac{e^{2}}{2C}=\frac{{(1.602\times{10}^{-19})}^{2}}{2\times29.51\times{10}^{-18}}=4.348\times{10}^{-22} J=2.713\mathrm{meV}$ (2)

In other words, a voltage $V>V_{thresh}$ must be applied to prevent significant Coulomb blockade effects. Moreover, Coulomb blockade is primarily noticeable at low voltages and low temperatures. In this study, the applied bias voltages are $V_{thresh}=0.5 V and 0.25 V$, while $V_{thresh}>2.713 \mathrm{mV}$, which is far above the low-voltage range. Additionally, the in-situ characterization was conducted at room temperature, making the Coulomb blockade effect induced by capacitance negligible.
